# Severe neonatal long QT syndrome type 3 treated with genotype-directed therapy and early left cardiac sympathetic denervation: a case report

**DOI:** 10.1093/ehjcr/ytag641

**Published:** 2026-08-27

**Authors:** Andrea Greco, Estefanía Martínez-Barrios, Agustín Lujan, Óscar Campuzano Larrea, Georgia Sarquella-Brugada

**Affiliations:** Arrhythmias, Inherited Cardiac Diseases and Sudden Death Unit, Hospital Sant Joan de Déu, Passeig Sant Joan de Déu 2, Esplugues de Llobregat, Barcelona 08950, Spain; Arrítmies Pediàtriques, Cardiologia Genètica i Mort Sobtada, Malalties Cardiovasculars en el Desenvolupament, Institut de Recerca Sant Joan de Déu (IRSJD), Santa Rosa 39-57, Esplugues de Llobregat, Barcelona 08950, Spain; European Reference Network for Rare, Low Prevalence and Complex Diseases of the Heart: ERN GUARD-Heart, Meibergdreef 9, 1105, Amsterdam, The Netherlands; Department of Pediatrics, Faculty of Medicine and Health Sciences, Universitat de Barcelona, Carrer de Casanova, 143, Barcelona 08036, Spain; Arrhythmias, Inherited Cardiac Diseases and Sudden Death Unit, Hospital Sant Joan de Déu, Passeig Sant Joan de Déu 2, Esplugues de Llobregat, Barcelona 08950, Spain; Arrítmies Pediàtriques, Cardiologia Genètica i Mort Sobtada, Malalties Cardiovasculars en el Desenvolupament, Institut de Recerca Sant Joan de Déu (IRSJD), Santa Rosa 39-57, Esplugues de Llobregat, Barcelona 08950, Spain; European Reference Network for Rare, Low Prevalence and Complex Diseases of the Heart: ERN GUARD-Heart, Meibergdreef 9, 1105, Amsterdam, The Netherlands; Arrhythmias, Inherited Cardiac Diseases and Sudden Death Unit, Hospital Sant Joan de Déu, Passeig Sant Joan de Déu 2, Esplugues de Llobregat, Barcelona 08950, Spain; Arrítmies Pediàtriques, Cardiologia Genètica i Mort Sobtada, Malalties Cardiovasculars en el Desenvolupament, Institut de Recerca Sant Joan de Déu (IRSJD), Santa Rosa 39-57, Esplugues de Llobregat, Barcelona 08950, Spain; European Reference Network for Rare, Low Prevalence and Complex Diseases of the Heart: ERN GUARD-Heart, Meibergdreef 9, 1105, Amsterdam, The Netherlands; Medical Sciences Department, School of Medicine, Universitat de Girona, Carrer Emili Grahit, 77, Girona 17003, Spain; Enfermedades Cardiovasculares, Centro de Investigación Biomédica en Red, Av. Monforte de Lemos, 3-5, Madrid 28029, Spain; Arrhythmias, Inherited Cardiac Diseases and Sudden Death Unit, Hospital Sant Joan de Déu, Passeig Sant Joan de Déu 2, Esplugues de Llobregat, Barcelona 08950, Spain; Arrítmies Pediàtriques, Cardiologia Genètica i Mort Sobtada, Malalties Cardiovasculars en el Desenvolupament, Institut de Recerca Sant Joan de Déu (IRSJD), Santa Rosa 39-57, Esplugues de Llobregat, Barcelona 08950, Spain; European Reference Network for Rare, Low Prevalence and Complex Diseases of the Heart: ERN GUARD-Heart, Meibergdreef 9, 1105, Amsterdam, The Netherlands; Department of Pediatrics, Faculty of Medicine and Health Sciences, Universitat de Barcelona, Carrer de Casanova, 143, Barcelona 08036, Spain; Medical Sciences Department, School of Medicine, Universitat de Girona, Carrer Emili Grahit, 77, Girona 17003, Spain

**Keywords:** Left cardiac sympathetic denervation, Implanted loop recorder, Long QT syndrome, Case report

## Abstract

**Background:**

Long QT syndrome type 3 may present in foetal or neonatal life with QT prolongation, 2:1 atrioventricular (AV) block, and life-threatening ventricular arrhythmias. Early recognition and individualizes genotype-directed management may be lifesaving.

**Case summary:**

A neonate delivered after foetal bradycardia and intermittent 2:1 AV block presented with severe QT prolongation and recurrent *torsades de pointes*. The functional 2:1 AV block persisted despite beta-blockade and magnesium administration. Because of recurrent ventricular arrhythmias, video-assisted thoracoscopic left cardiac sympathetic denervation (LCSD) and implantable loop recorder were performed on Day 8 of life. Genetic testing identified a *de novo* likely pathogenic *SCN5A*_p.(Ala1656Val) missense variant. Subsequent lidocaine testing demonstrated marked QT shortening, supporting sodium channel-mediated disease and guiding treatment with mexiletine and flecainide. Long-term follow-up required implantable cardioverter-defibrillator therapy, but arrhythmic burden progressively improved.

**Discussion:**

This case supports the potential role of early multimodal treatment in malignant neonatal long QT syndrome type 3, combining LCSD, continuous rhythm monitoring, and genotype-directed sodium channel blockade.

Learning points

- Fetal bradycardia with intermittent 2:1 AVB may indicate malignant neonatal long QT syndrome type 3 and warrants urgent postnatal rhythm assessment.
- Early left cardiac sympathetic denervation and continuous rhythm monitoring may benefit neonates with recurrent torsades de pointes despite medical therapy.
- Lidocaine testing and genetic diagnosis may confirm sodium channel disease and guide targeted treatment with mexiletine and flecainide.

## Introduction

Congenital long QT syndrome type 3 (LQTS3) is caused by pathogenic variants in the *SCN5A* gene leading to increased late sodium current and delayed ventricular repolarization. Neonatal presentation is rare but may be severe, particularly when associated with fetal bradycardia, functional 2:1 atrioventricular (AV) block, and *torsades de pointes* (TdP). These cases require rapid diagnosis and individualized treatment, including beta-blockade, sodium channel blockade, left cardiac sympathetic denervation (LCSD), continuous rhythm monitoring, and, in selected patients, implantable cardioverter-defibrillator (ICD) therapy. We report a neonate with malignant LQTS3 associated with a novel *de novo* likely pathogenic *SCN5A* missense variant, managed with early LCSD, continuous rhythm monitoring, and genotype-directed sodium channel blockade.^1^ This case provides long-term follow-up of an individualized multimodal strategy initiated during the neonatal period.

## Summary figure

**Figure:**
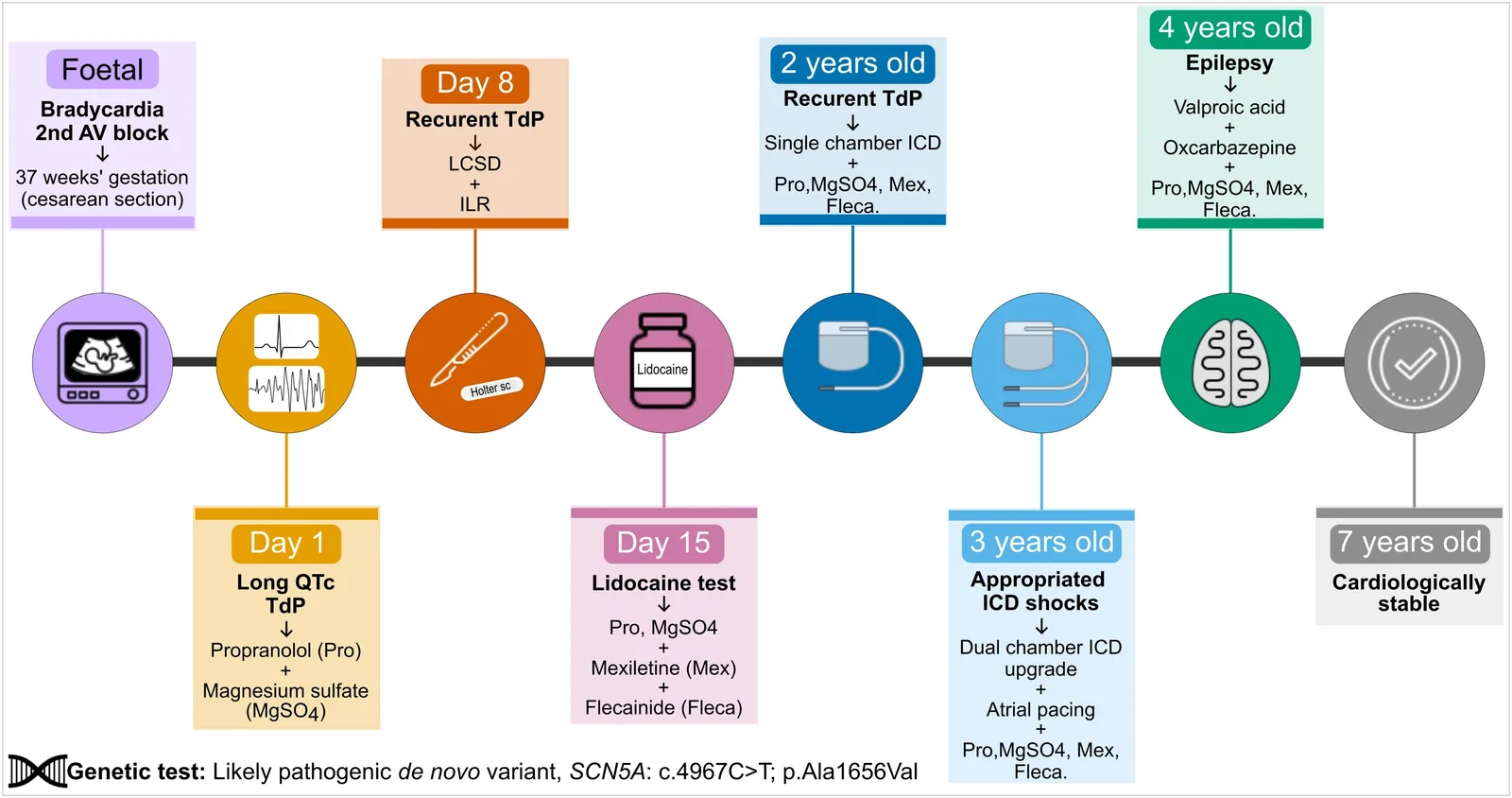


## Case presentation

A 40-year-old pregnant woman was referred at 36 weeks’ gestation because of persistent fetal bradycardia of 65–70 b.p.m., intermittent 2:1 AV block, and mild pericardial effusion. No structural cardiac abnormality was identified. Because an aggressive inherited arrhythmia syndrome was suspected, immediate delivery was planned and performed via caesarean section at 37 + 1 weeks’ gestation. The neonate weighed 3.2 kg at birth and Apgar scores of 9 at both 1 and 5 min.

Immediately after birth, the neonate presented with severe bradycardia of ∼50 b.p.m. with alternating 2:1 and 1:1 AV conduction. A 12-lead electrocardiogram demonstrated marked QT prolongation, with a QT interval of ∼880 ms and a Bazett-corrected QT interval of approximately 820 ms (*Figure 1*). Continuous rhythm monitoring showed recurrent episodes of polymorphic ventricular tachycardia consistent with TdP. Physical examination was otherwise unremarkable. echocardiography showed normal cardiac anatomy and function, and serum electrolytes, thyroid function, and metabolic screening were normal.

**Figure 1:**
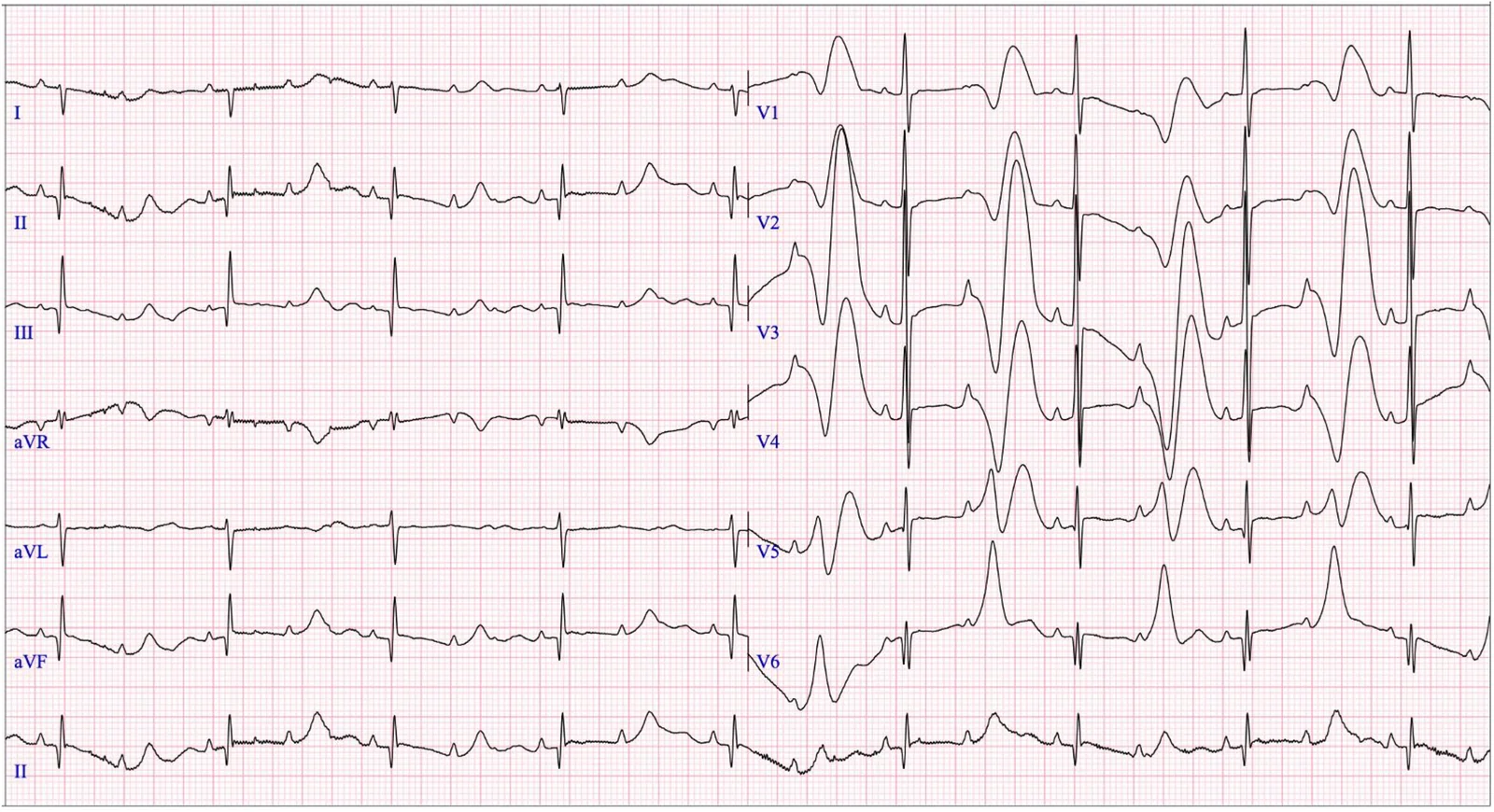
Electrocardiogram at birth with sinus rhythm with heart rhythm of 50–55 bpm. 2:1 atrioventricular block. *(A)* QT 880 ms, *(B)* QTc 820 ms. The 2:1 atrioventricular block is a unique manifestation of this type of long QT syndrome. Extreme prolongation of the ventricular refractory period can lead to a pseudo-2:1 atrioventricular block.

Treatment with propranolol was started immediately after birth (1 mg/kg/day, divided every 8 h). Because recurrent TdP persisted, intravenous magnesium (up to 50 mg/kg IV) and continuous esmolol infusion were added (up to 100 µg/kg/min). Despite intensified beta-blockade and magnesium supplementation, the infant continued to have recurrent ventricular arrhythmias, raising concern for an electrical storm. Given the persistence of life-threatening ventricular arrhythmias despite medical therapy, the limitations of transvenous pacing and ICD implantation in a neonate, and the need for rapid arrhythmic stabilization, LCSD was selected as an adjunctive treatment. On Day 8 of life, video-assisted thoracoscopic LCSD was performed, with removal of the lower half of the left stellate ganglion and thoracic ganglia T2–T4. A subcutaneous implantable loop recorder (ILR, Reveal LINQ™, model LNQ11; Medtronic), the smallest commercially available device at the time, was inserted during the same procedure to enable continuous remote rhythm surveillance (*Figure 2*). The intervention was well tolerated, with no acute complications. Ventricular ectopy decreased markedly after surgery, and no sustained ventricular tachycardia occurred in the following 48 h.

**Figure 2:**
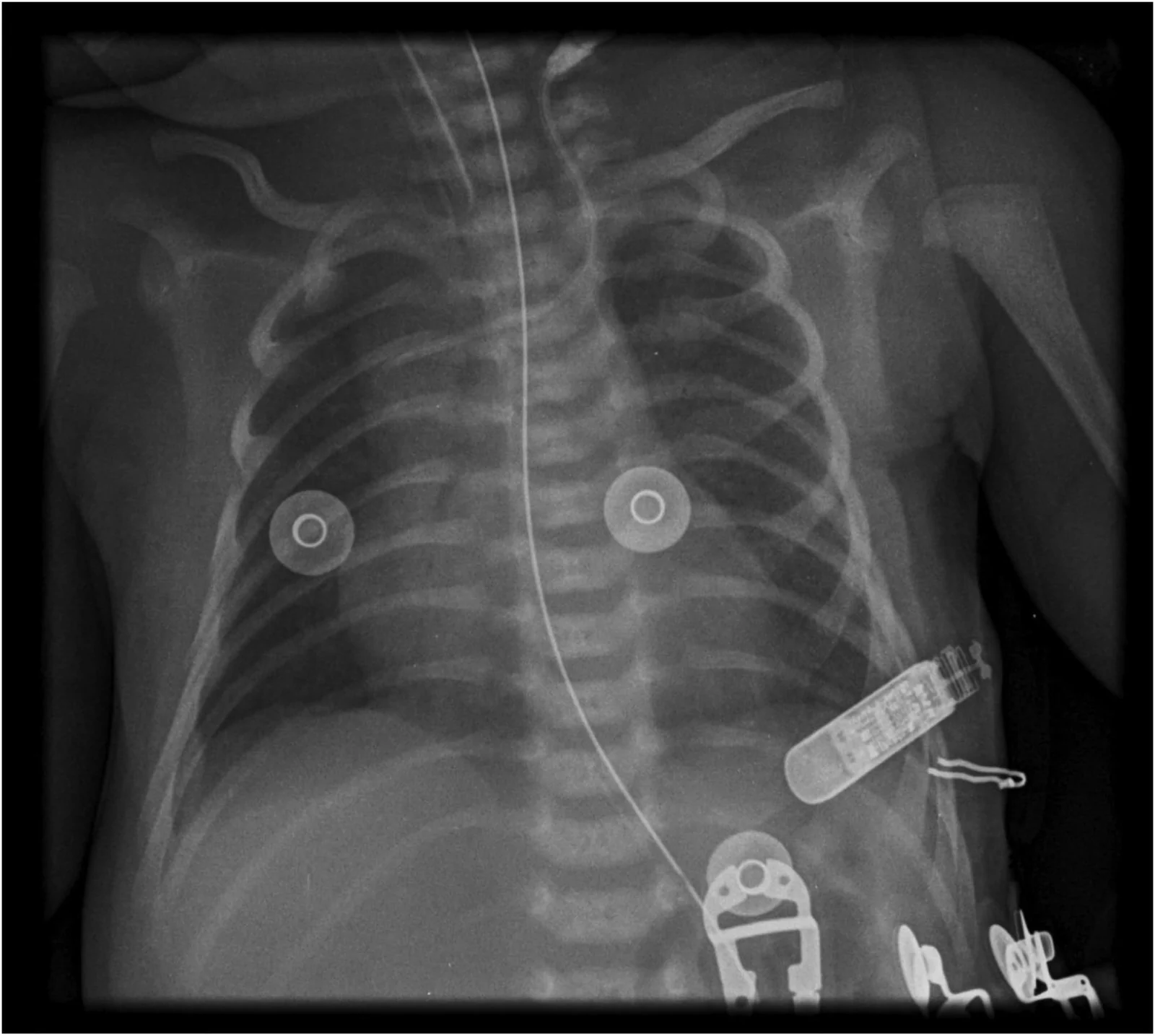
Chest X-ray after left cardiac sympathetic denervation and implantable loop recorder implantation.

A lidocaine challenge produced significant QT shortening, consistent with sodium channel-mediated QT prolongation (*Figure 3* and *Figure 4*). Mexiletine was therefore initiated (up to 20 mg/kg/day), followed by flecainide (up to 5 mg/kg/day) for additional sodium channel blockade. The patient was discharged on Day 30 of life on propranolol (2 mg/kg/day), mexiletine (20 mg/kg/day), flecainide (up to 5 mg/kg/day), and magnesium supplementation, with remote rhythm monitoring through the ILR. Genetic testing, including a comprehensive panel of genes associated with inherited channelopathies, identified a heterozygous *de novo* missense variant in *SCN5A*, NM_198056.3: c.4967C>T, p.(Ala1656Val), affecting a conserved residue in the Nav1.5 inactivation gate. The variant was absent from the Genome Aggregation Database (gnomAD v4.1.1) and was classified as likely pathogenic according to the ACMG/AMP framework (PS2_Strong, PM1_Moderate, PM2_supporting, and PP3_supporting criteria).^2^ The amino acid Alanine (Ala, A: non-polar Aliphatic) is substituted by amino acid Valine (Val, V: non-polar Aliphatic essential). Despite similar structure, *in silico* prediction also supports pathogenicity, including REVEL (score of 0.941) and AlphaMissense (Score of 0.969). This is due to the presence of two additional carbon–hydrogen groups in the side chain of the amino acid Val, which imparts greater non-polar character, making it significantly more hydrophobic than the amino acid Ala.

**Figure 3:**
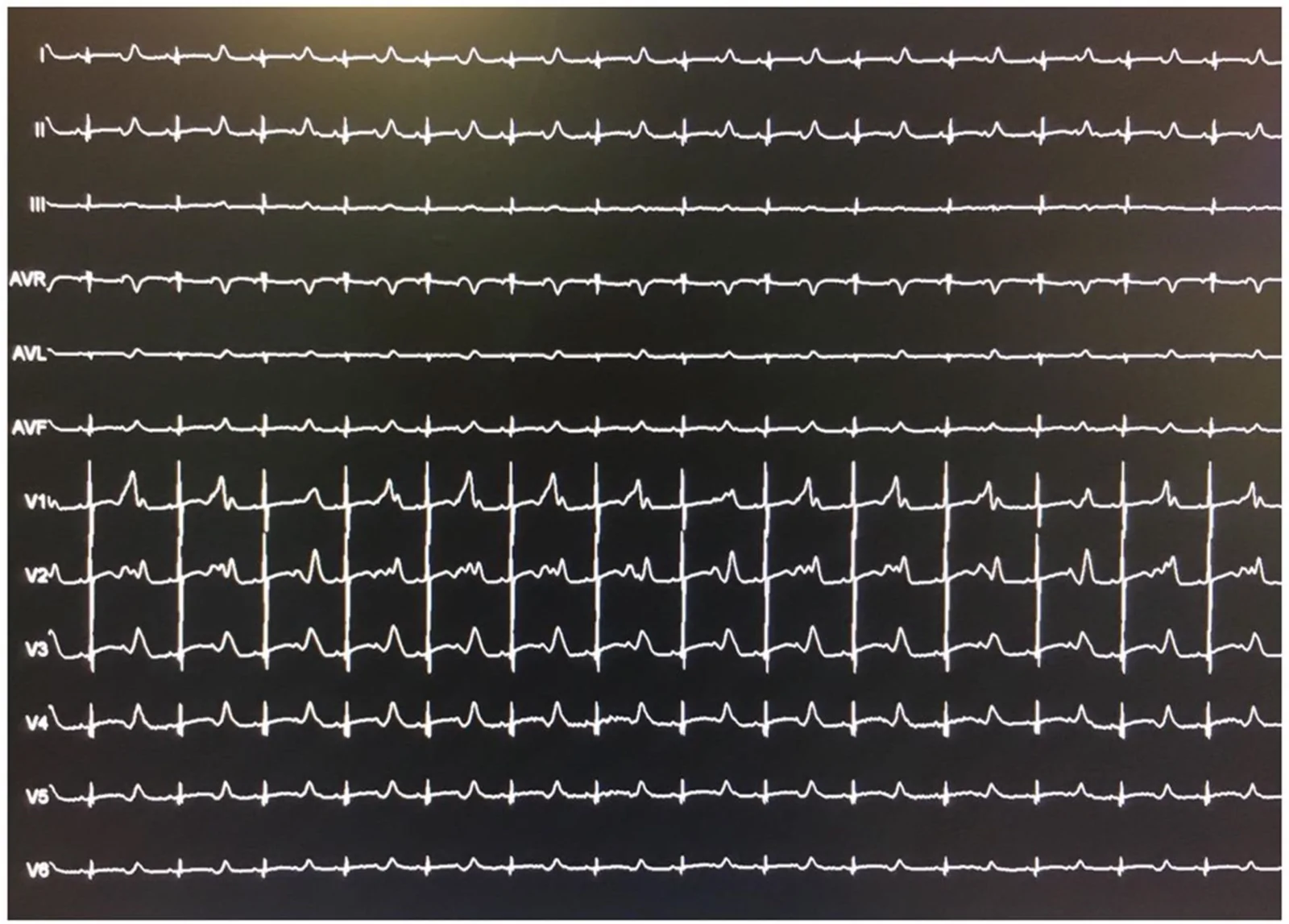
Electrocardiogram before lidocaine infusion. Sinus rhythm with functional 2:1 atrioventricular block.

**Figure 4:**
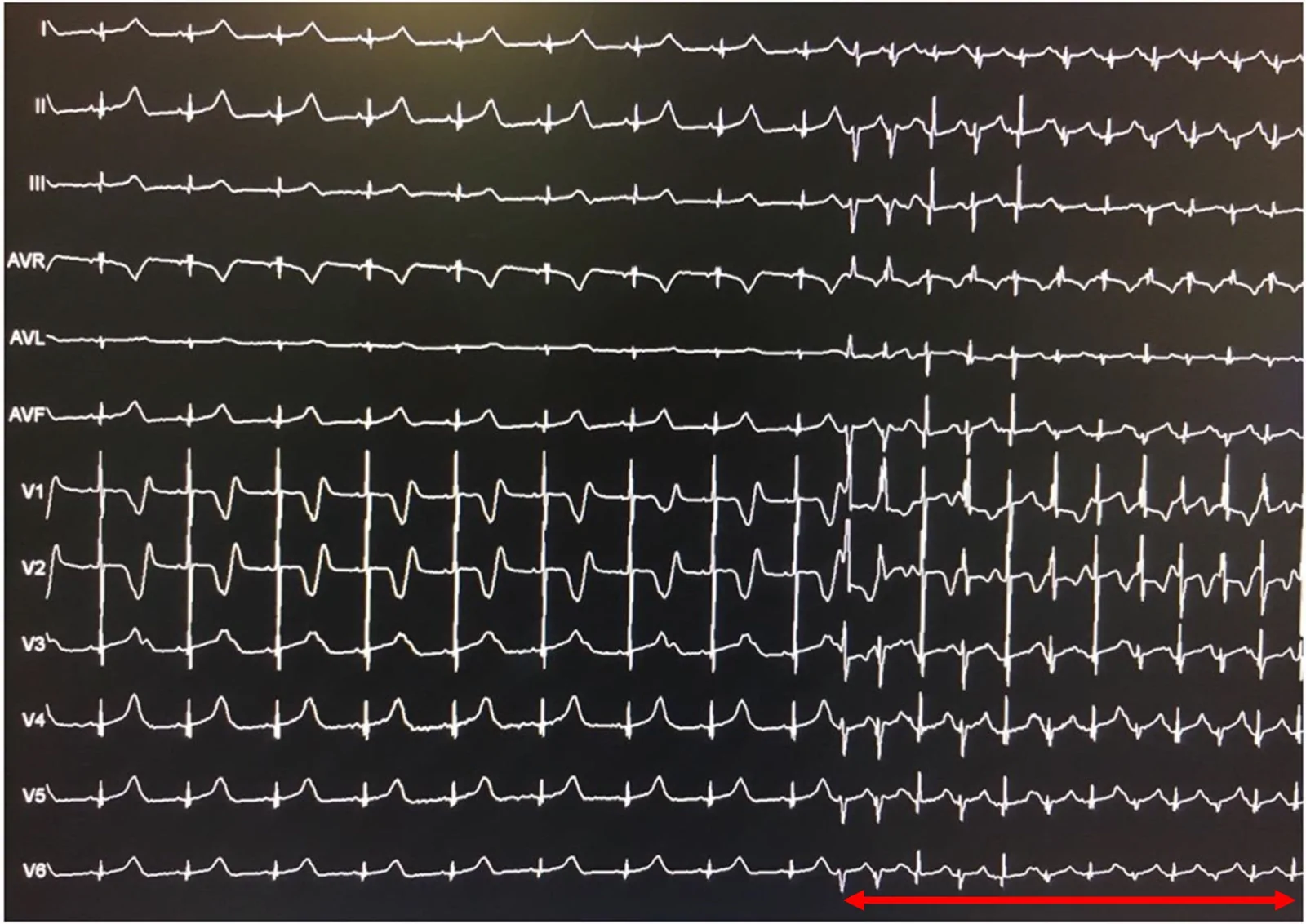
Electrocardiogram after lidocaine infusion. Transition between the previous electrocardiogram and sinus rhythm with 1:1 atrioventricular conduction secondary to a shortening of >50 ms of QT interval (red arrows).

During follow-up, the ILR documented isolated episodes of non-sustained ventricular tachycardia and self-terminating TdP (*Figure 5*). Because of persistent high arrhythmic risk, a single-chamber ICD was implanted at 2 years of age. The device subsequently delivered appropriate therapies for ventricular tachycardia and was later upgraded to a dual-chamber system at 3 years of age, allowing atrial pacing support and optimization of beta-blocker therapy. At 4 years of age, the child developed epilepsy in the absence of other neurological, neuromuscular, or dysmorphic features, which was considered potentially related to sodium-channel dysfunction. At last follow-up, aged 7 years, he remained clinically stable on mexiletine, flecainide, propranolol, and magnesium supplementation, with no recent ICD therapies and a QTc consistently below 500 ms.

**Figure 5:**
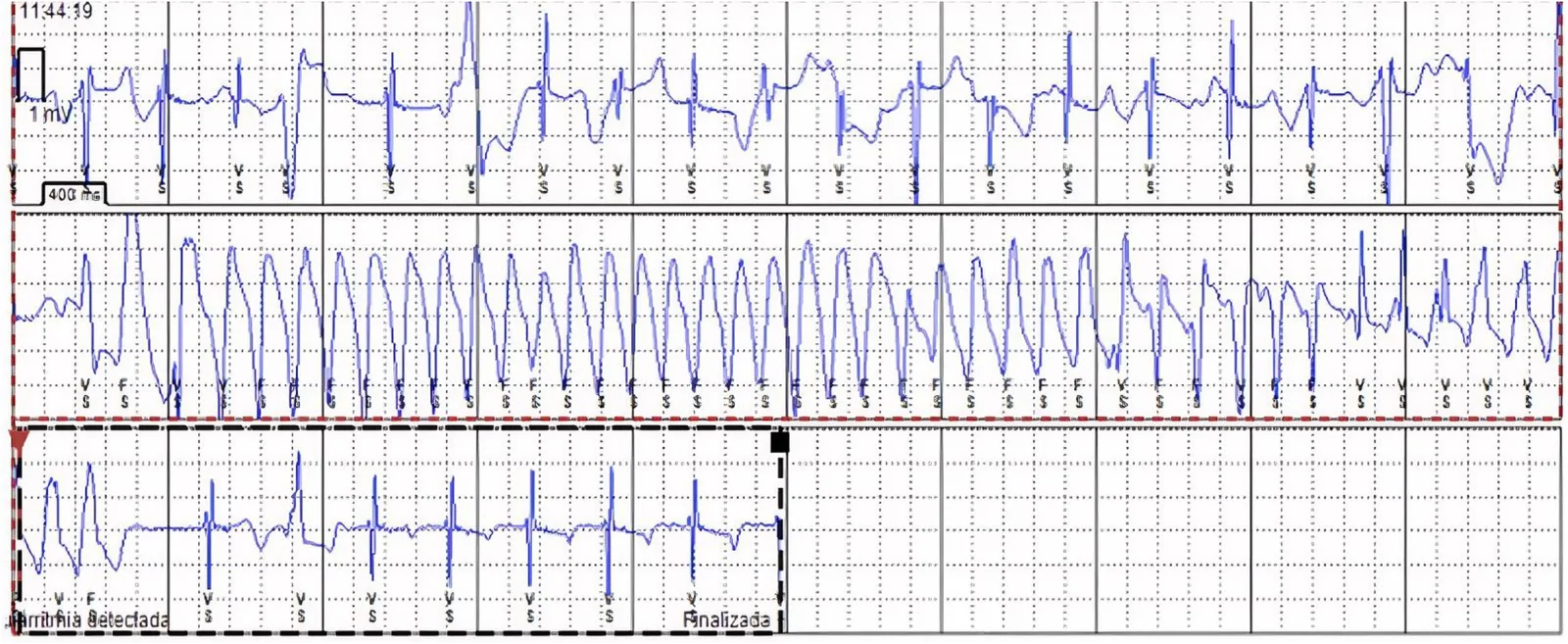
Implantable loop recorder telemetry: self-limited episode of Torsade de pointes preceded by frequent premature ventricular contractions with different morphologies and lasting with complete recovery of sinus rhythm with prolonged QT and a premature ventricular contraction.

## Discussion

This case underscores the aggressive management required in neonatal LQTS3. Fetuses/neonates with *SCN5A* deleterious variants and 2:1 AV block face a very high risk of lethal arrhythmias. In a multicenter LQTS registry, LQTS3 neonates had the worst outcomes compared to other types of LQTS.^3^ In LQTS3, arrhythmic events are frequently bradycardia-triggered, which helps explain breakthrough TdP under β-blocker therapy and supports adjunctive strategies such as LCSD and genotype-directed sodium-channel blockade.^4^

Current HRS/EHRA/APHRS consensus guidelines recommend LCSD in high-risk LQTS when β-blockade fails.^1,5,6^ LCSD acutely withdraws sympathetic input to the heart, raising the threshold for ventricular arrhythmia. To our knowledge, this represents the smallest infant reported to have undergone LCSD to date. In our case, LCSD effectively suppressed TdP storms, aligning with reports of LCSD as an ‘acute therapy’ in paediatric LQTS unresponsive to drugs.^6^ Continuous monitoring by ILR was pivotal; it safely documented transient ectopy (prompting outpatient management adjustments) without patient discomfort. A recent report similarly showed successful loop recorder use in a 2.7 kg LQTS3 infant to detect breakthrough arrhythmias.^7^

Once arrhythmias were controlled following LCSD, and the causal genetic variant was identified, we could safely tailor genotype-specific therapy. Although the *SCN5A*_p.Ala1656Val variant is novel, a different rare variant affecting the same residue (p.Ala1656Asp) was reported in a neonate with severe LQTS3 and demonstrated gain-of-function effects. Notably, mexiletine was the only therapy able to normalize channel inactivation kinetics in that case.^8^ These findings provide indirect support for a similar effect of p.(Ala1656Val) on Na ^+^ -channel inactivation. However, because functional data are available only for a different substitution at the same residue, the proposed mechanism remains hypothetical pending variant-specific functional validation.^4^ The acute lidocaine test confirmed profound QT shortening (by abolishing late I_Na_) and guided initiation of mexiletine. Flecainide was added because QT prolongation persisted despite mexiletine, with further QT shortening observed. Treatment was individualized according to the electrocardiographic response, given the mutation-specific variability in sodium channel blocker efficacy. This stepwise, genotype-guided pharmacotherapy contrasts with empiric β-blocker escalation and likely improved outcomes.

Our management strategy is consistent with contemporary expert guidance, which supports timely escalation, including LCSD and, when indicated, ICD, in high-risk LQTS with recurrent events despite optimized medical treatment, a scenario that is particularly relevant in malignant neonatal LQTS3 presentations.^3^ Notably, even combined strategies may be imperfect: registry data show LCSD only reduced arrhythmia burden in fewer than 50% of neonatal LQTS3 cases, and some still suffered fatal events despite LCSD and ICD.^3^ Accordingly, care should be individualized within a multidisciplinary framework with readiness to escalate and adjust therapies as the clinical trajectory evolves. Within this framework, continuous remote rhythm surveillance via ILR in a very low-weight infant is a pragmatic adjunct that can detect breakthrough arrhythmias and guide outpatient titration and safety monitoring.^7,9^

This case supports the potential role of an early multimodal strategy for malignant neonatal LQTS3, combining rapid phenotypic recognition, genetic diagnosis, provocative sodium channel-blocker testing, LCSD, continuous rhythm surveillance, and timely ICD therapy when clinically indicated, while acknowledging that these observations derive from a single case.

## Patient perspective

The patient’s parents expressed gratitude for the multidisciplinary care received and emphasized the importance of clear communication during the neonatal period, when urgent decisions regarding surgery, genetic testing, and long-term rhythm management were required.

## Lead author biography


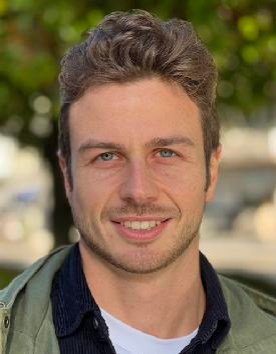


Paediatric cardiologist and electrophysiologist at Sant Joan de D=C3=A9u Barcelona Children=E2=80=99s Hospital, with expertise in complex paediatric arrhythmias and inherited cardiomyopathies. He is currently pursuing a PhD at the University of Barcelona focused on advances in paediatric arrhythmias and inherited cardiomyopathies. His work integrates clinical care, research, and innovation in paediatric electrophysiology. He is an active member of the AEPC and other scientific societies, author of more than 15 peer-reviewed publications in indexed journals, and serves as Councillor of the AEPC Working Group on Sports Cardiology and Coordinator of the Pediatric Cardiology Working Group of the Catalan Society of Pediatrics.

## Data Availability

All data are available in the manuscript.

## References

[1] Zeppenfeld K, Tfelt-Hansen J, de Riva M, Winkel BG, Behr ER, Blom NA, et al 2022 ESC guidelines for the management of patients with ventricular arrhythmias and the prevention of sudden cardiac death. Eur Heart J 2022;43:3997–4126.

[2] Richards S, Aziz N, Bale S, Bick D, Das S, Gastier-Foster J, et al Standards and guidelines for the interpretation of sequence variants: a joint consensus recommendation of the American College of Medical Genetics and Genomics and the Association for Molecular Pathology. Genet Med 2015;17:405–424.

[3] Moore JP, Gallotti RG, Shannon KM, Bos JM, Sadeghi E, Strasburger JF, et al Genotype predicts outcomes in fetuses and neonates with severe congenital long QT syndrome. JACC Clin Electrophysiol 2020;6:1561–1570.

[4] Pérez-Riera AR, Barbosa-Barros R, Daminello Raimundo R, da Costa de Rezende Barbosa MP, Esposito Sorpreso IC, de Abreu LC. The congenital long QT syndrome type 3: an update. Indian Pacing Electrophysiol J 2018;18:25–35.

[5] Priori SG, Wilde AA, Horie M, Cho Y, Behr ER, Berul C, et al HRS/EHRA/APHRS expert consensus statement on the diagnosis and management of patients with inherited primary arrhythmia syndromes: document endorsed by HRS, EHRA, and APHRS in May 2013 and by ACCF, AHA, PACES, and AEPC in June 2013. Heart Rhythm 2013;10:1932–1963.

[6] Shortland J, Marquez D, Al-Radi O, Sanatani S. Left cardiac sympathetic denervation as an acute treatment of torsades in a paediatric case of long QT. CJC Pediatr Congenit Heart Dis 2023;2:57–60.

[7] Wong A, Cortez D. Implantable loop recorder via subscapular approach in 2.7 kg neonate. Future Cardiol 2024;20:689–693.

[8] Kim H-J, Kim B-G, Park JE, Ki C-S, Huh J, Youm JB, et al Characterization of a novel LQT3 variant with a selective efficacy of mexiletine treatment. Sci Rep 2019;9:12997.

[9] Aziz PF, Tanel RE, Zelster IJ, Pass RH, Wieand TS, Vetter VL, et al Congenital long QT syndrome and 2:1 atrioventricular block: an optimistic outcome in the current era. Heart Rhythm 2010;7:781–785.

